# Lipid metabolism, viral infection, and antiviral immunity: a new host-pathogen interface

**DOI:** 10.3389/fcimb.2026.1765502

**Published:** 2026-03-05

**Authors:** Li Jun Yang, Min Tang, Le Yang

**Affiliations:** 1 Department of Pharmacy, Yiyang Medical College, Yiyang, China; 2 Department of Gastroenterology, Yiyang Central Hospital, Yiyang, China

**Keywords:** antiviral immunity, host-pathogen interface, immune regulation, lipid metabolism, viral infection

## Abstract

Viral infections pose significant challenges to global health. Lipid metabolism plays a crucial role in various biological processes, including cell membrane structure, signaling, and energy homeostasis. Recent studies have highlighted the intricate relationship between lipid metabolism and viral infections, revealing how viruses exploit host lipid pathways to facilitate their replication and assembly. This review aims to elucidate the mechanisms by which viruses manipulate lipid metabolism and the subsequent impact on antiviral immunity. We systematically analyze the biological basis of lipid synthesis and degradation, emphasizing the role of lipids in immune cell function and the regulation of antiviral responses. Furthermore, we explore how altered lipid metabolism can influence immune responses in disease states, providing insights into the differential utilization of lipid pathways by various viruses. This review highlights suggest potential therapeutic strategies, including the development of antiviral drugs targeting lipid metabolism, modulation of lipid pathways to enhance immune responses, and combination therapies that integrate lipid metabolism modulation with conventional antiviral treatments. Future research directions are proposed, focusing on the interaction between lipid metabolism and emerging viral strains, the application of metabolomics in viral infection studies. This comprehensive review underscores the significance of lipid metabolism as a novel host-pathogen interface, paving the way for innovative therapeutic approaches in combating viral infections.

## 1 Introduction

The intricate relationship among lipid metabolism, viral infections, and antiviral immunity has garnered increasing attention in recent years, particularly as our understanding of host-pathogen interactions deepens. Lipids serve as essential components in cellular structures, energy storage, and signaling pathways, making them pivotal for both viral replication and the host’s immune response. Viruses, which lack their own metabolic machinery, rely heavily on the host cell’s lipid metabolism to facilitate their life cycle, including entry, replication, and egress. This review aims to elucidate the complex interplay between lipid metabolism and viral infections, emphasizing how alterations in lipid pathways can influence the efficiency of antiviral immune responses.

Recent studies have demonstrated that enveloped viruses, such as Severe Acute Respiratory Syndrome Coronavirus 2 (SARS-CoV-2), exploit host lipids at various stages of their life cycle. For instance, the viral envelope is derived from host cell membranes, and specific lipids are crucial for viral entry and replication (Alketbi et al., 2021). Dysregulation of lipid metabolism can create an environment conducive to viral proliferation, as seen in infections like Corona Virus Disease 2019 (COVID-19), where altered lipid profiles have been associated with disease severity (Abu-Farha et al., 2020). The manipulation of lipid pathways by viruses not only aids their replication but can also modulate the host immune response, potentially leading to an impaired ability to combat the infection effectively (Requena et al., 2025).

The role of lipid metabolism in antiviral immunity is multifaceted. Lipids are involved in the production of signaling molecules that can enhance or inhibit immune responses. For instance, several classes of lipid mediators, including eicosanoids and sphingolipids, produced during viral infections can modulate inflammation and influence the activation of immune cells (Theken et al). The interplay between lipid metabolism and immune signaling pathways is critical; for example, the activation of nuclear factor erythroid 2-related factor 2 (NRF2) has been shown to regulate both lipid metabolism and antiviral responses, highlighting the need for a balanced lipid environment to support effective immune function (Havranek et al., 2021). Moreover, the impact of metabolic reprogramming during viral infections is significant. Viruses can induce changes in the host’s metabolic pathways, leading to increased lipid synthesis and altered energy utilization, which in turn supports viral replication (Chu et al., 2024). This metabolic hijacking can also affect the innate immune responses, as immune cells adapt their metabolism to generate energy and biosynthetic precursors necessary for an effective antiviral response (Li et al., 2023). Understanding these metabolic alterations provides insights into potential therapeutic strategies that target lipid metabolism to enhance antiviral immunity.

In summary, the relationship among lipid metabolism, viral infections, and antiviral immunity is a dynamic and complex interplay that is crucial for both viral pathogenesis and host defense. As research in this area continues to evolve, it becomes increasingly clear that targeting lipid metabolic pathways could offer novel therapeutic approaches to combat viral infections and enhance immune responses. This review will delve deeper into the mechanisms by which lipid metabolism influences viral infections and the host’s immune response, providing a comprehensive understanding of this emerging field.

## 2 Basic mechanisms of lipid metabolism

### 2.1 Biological basis of lipid synthesis and degradation

Lipid metabolism is a complex biochemical process that encompasses the synthesis and degradation of lipids, which are essential for various cellular functions. The synthesis of lipids, known as lipogenesis, primarily occurs in the liver and adipose tissue, where fatty acids are synthesized from acetyl-CoA through a series of enzymatic reactions. Key enzymes involved in this process include fatty acid synthase and acetyl-CoA carboxylase, which catalyze the conversion of acetyl-CoA to malonyl-CoA and subsequently to long-chain fatty acids. Conversely, lipid degradation, or lipolysis, involves the breakdown of triglycerides into glycerol and free fatty acids, primarily facilitated by hormone-sensitive lipase and adipose triglyceride lipase. This process is crucial for energy production, especially during periods of fasting or increased energy demand. Dysregulation of lipid metabolism can lead to metabolic disorders such as obesity, diabetes, and cardiovascular diseases, highlighting the importance of understanding these pathways in maintaining metabolic health (Chandel, 2021; Khan et al., 2022; Raal and Cuchel, 2023).

### 2.2 Role of lipids in cell membrane structure

Lipids play a fundamental role in forming the structural basis of cellular membranes, primarily as phospholipids, cholesterol, and sphingolipids. The phospholipid bilayer forms the core structure of cell membranes, providing fluidity and flexibility essential for membrane integrity and function (Isik and Cizmecioglu). Cholesterol, interspersed within the phospholipid bilayer, modulates membrane fluidity and stability (Minto et al., 2004), while sphingolipids contribute to membrane microdomains known as lipid rafts, which are critical for cell signaling and protein sorting (Bagnat et al., 2000). These membranes not only serve as barriers to separate cellular compartments but also facilitate communication between cells and their environment through receptor-mediated signaling. The composition of membrane lipids can significantly influence cellular processes such as signal transduction, cell recognition, and membrane trafficking, underscoring their vital role in cellular physiology (Keshavarz et al., 2020; Ali and Szabó, 2023; Qu et al., 2023).

### 2.3 Importance of lipid signaling in cellular functions

Lipid signaling is a crucial aspect of cellular function, influencing various physiological processes such as inflammation (Michalik and Wahli, 2008), cell growth (Hannun and Obeid, 2008), and apoptosis (Jonassen et al., 2003). Lipids act as signaling molecules that can activate specific pathways through their metabolites. For instance, phosphoinositides, derived from phosphatidylinositol, play a pivotal role in cell signaling by recruiting proteins to membranes and activating downstream signaling cascades (Echard, 2012). Additionally, bioactive lipids such as prostaglandins and leukotrienes, derived from arachidonic acid, are involved in inflammatory responses and immune regulation (Woszczek et al., 2003; Esser-von Bieren, 2017; Taketomi and Murakami, 2017). The dysregulation of lipid signaling pathways has been implicated in numerous diseases, including cancer and metabolic disorders, highlighting the necessity for a deeper understanding of lipid signaling mechanisms in health and disease management (Long et al., 2018; Liu et al., 2024). Therefore, lipid metabolism encompasses a wide array of biological processes critical for maintaining cellular structure and function. Understanding the intricate mechanisms of lipid synthesis, degradation, and signaling is essential for elucidating their roles in health and disease. Further research in this area may provide insights into potential therapeutic targets for metabolic and inflammatory diseases, enhancing our ability to manage these conditions effectively.

## 3 How viruses exploit host lipid metabolism

**Figure 1:**
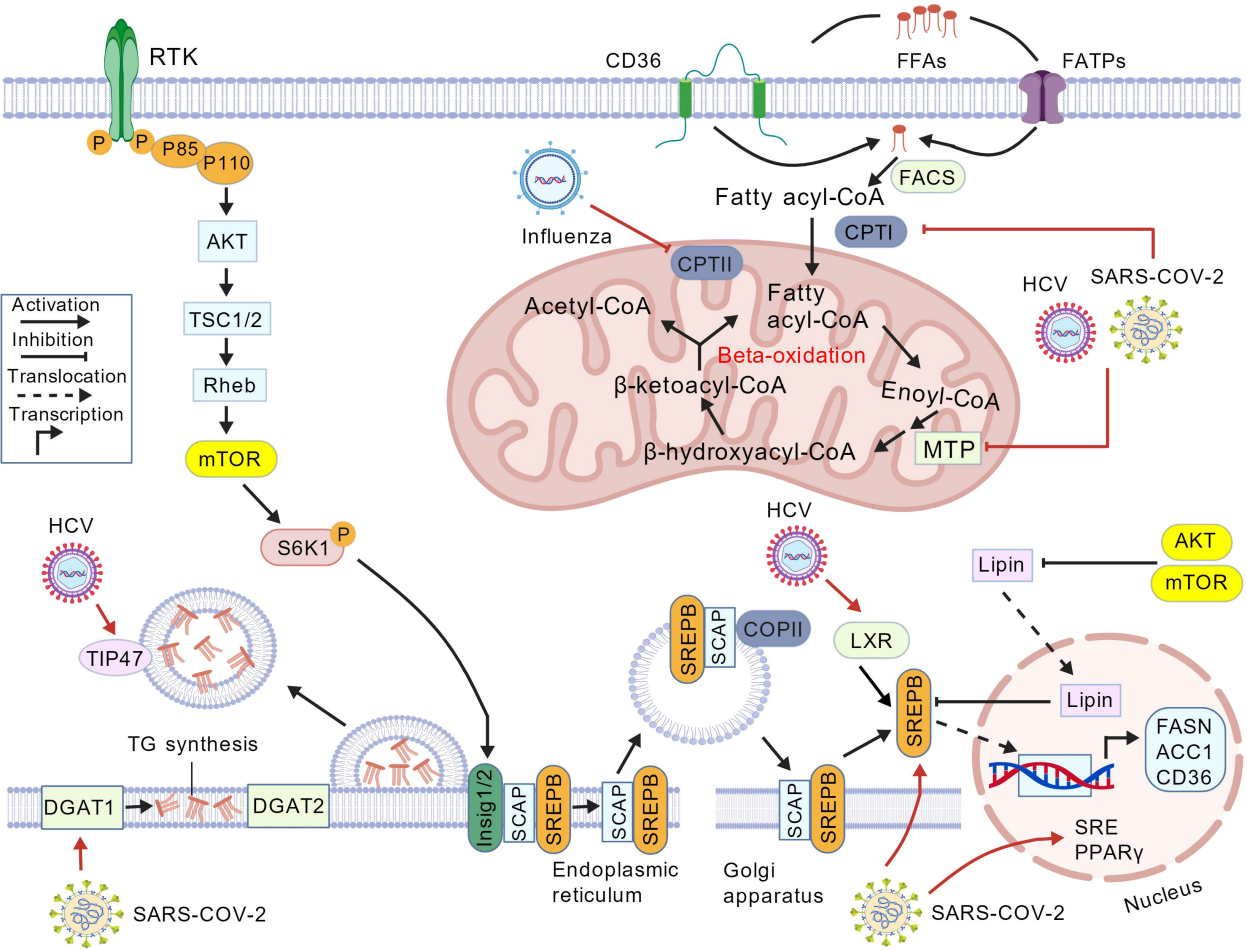
Lipid metabolism and related pathways are altered by viruses. Upon infection, there is an increase in lipid synthesis, which is mediated by increasing the activity of main fatty acid synthesis enzymes such as FASN and ACC and transcription factors such as SREBP. Moreover, this figure also represents virus modulation of beta-oxidation and lipid droplet formation. ACC, acetyl-CoA carboxylase; FASN, fatty acid synthase; COPII, coat protein complex II; DGAT, diacylglycerol O-acyltransferase; FACS, fatty acyl-CoA synthetase; FATP, fatty acid transport protein; CPT, carnitine palmitoyltransferase; FFA, free fatty acid; HCV, hepatitis C virus; LXR, liver X receptor; mTOR, mechanistic target of rapamycin; MTP, mitochondrial trifunctional protein; P, phosphate group; PPARγ, peroxisome proliferator-activated receptor γ; Rheb, Ras homolog enriched in brain; TSC, tuberous sclerosis complex; RTK, receptor tyrosine kinase; S6K1, S6 kinase beta-1; SCAP, SREBP cleavage-activating protein; SRE, sterol regulatory element; SREBP, sterol regulatory element-binding protein; TG, triacylglycerol; Created with BioGDP.com.

Viruses are adept at manipulating host cellular processes to facilitate their replication and spread. One of the critical areas they exploit is lipid metabolism, which plays a pivotal role in various stages of the viral life cycle. By hijacking the host’s lipid synthesis pathways, viruses can create an environment conducive to their survival and proliferation. This section delves into the mechanisms through which viruses activate lipid synthesis pathways, the relationship between lipid metabolism and viral assembly and release, and the differential utilization of lipid metabolism by various viruses (**Figure 1**).

### 3.1 Mechanisms of virus-induced activation of lipid synthesis pathways

Viruses have evolved sophisticated strategies to activate host lipid synthesis pathways, which are crucial for their replication. For instance, enveloped viruses, such as coronaviruses (Alketbi et al., 2021) and hepatitis C virus (HCV) (Villareal et al), utilize host lipids to form their viral envelopes and replication compartments. The entry of enveloped viruses into host cells necessitates the fusion of viral and cellular membranes, a process that heavily relies on the lipid composition of these membranes (Ohnishi). Upon infection, viruses can induce significant alterations in host lipid metabolism, leading to increased synthesis of specific lipid species that are essential for viral replication and assembly.

One of the key mechanisms by which viruses activate lipid synthesis involves the modulation of host transcription factors and signaling pathways. For example, the viral protein non-structural 5A (NS5A) of HCV interacts with host factors to enhance the synthesis of lipids necessary for the formation of viral replication complexes (Vogt et al., 2013). Additionally, viruses can manipulate lipid droplet dynamics and promote the accumulation of neutral lipids, which serve as energy reserves and building blocks for viral particles (Farías et al., 2024). The activation of lipid synthesis pathways is not merely a byproduct of viral infection; rather, it is a strategic maneuver that viruses exploit to ensure their successful replication and egress from host cells. Furthermore, the interplay between viral proteins and host lipid metabolism is complex and multifaceted. For instance, the activation of the sterol regulatory element-binding proteins (SREBPs), which are key regulators of lipid metabolism, is often hijacked by viruses to enhance lipid synthesis. This activation leads to increased levels of fatty acids and triglycerides, which are crucial for viral assembly (Ma et al., 2024). By understanding these mechanisms, researchers can identify potential therapeutic targets that disrupt the viral exploitation of lipid metabolism, potentially leading to novel antiviral strategies.

### 3.2 Viral internalization: lipid-mediated membrane fusion

Viral internalization is a critical step in the viral life cycle, where viruses exploit host cellular machinery to gain entry into host cells. One of the primary mechanisms facilitating this process is lipid-mediated membrane fusion, a phenomenon that underscores the intricate relationship between viral pathogens and host lipid metabolism. Enveloped viruses, such as coronaviruses (Ou et al., 2016) and influenza viruses (Scheiffele et al., 1999), rely heavily on host lipids for their entry, replication, and egress. This dependence on lipid metabolism is not merely a passive interaction; rather, viruses actively manipulate host lipid pathways to create an environment conducive to their replication. The lipid bilayer of the host cell membrane serves as the first barrier to viral entry, and viruses have evolved sophisticated strategies to exploit this barrier.

The process of viral entry begins with the attachment of the virus to specific receptors on the host cell membrane. This initial interaction is often mediated by viral glycoproteins that recognize and bind to host cell receptors, which can include lipid rafts-cholesterol-rich microdomains that play a critical role in cell signaling and membrane trafficking (Casasnovas, 2024). Once bound, the virus induces conformational changes in its envelope proteins, leading to the fusion of the viral and cellular membranes. This is where the lipid composition of both the virus and the host cell becomes crucial. For instance, studies have shown that the lipid composition of the host cell membrane can significantly influence the efficiency of viral entry. Viruses such as the SARS-CoV-2 utilize specific lipids to facilitate membrane fusion, highlighting the importance of lipid interactions in the viral life cycle (Dadhich and Kapoor, 2020). Moreover, the manipulation of host lipid metabolism by viruses extends beyond mere entry. Once inside the host cell, viruses can hijack lipid metabolic pathways to support their replication. For example, during vesicular stomatitis virus (VSV) infection, alterations in the host lipid profile were observed, indicating that specific lipid species are crucial for viral replication and assembly (Cui et al., 2023). This reprogramming of lipid metabolism often involves the depletion of certain lipids, such as lysophosphatidylcholine (LPC), which can affect membrane curvature and serve as signaling molecules within the cell. Conversely, the accumulation of ceramide and sphingomyelin lipids during viral infection suggests that these lipids may play a role in the assembly of viral particles (Liu et al., 2023).

The interplay between viral infection and lipid metabolism is further complicated by the host’s immune response. The NRF2 has been identified as a key regulator of lipid metabolism during viral infections, influencing both the host’s metabolic pathways and the antiviral response (Muzammil et al). NRF2 activation can lead to changes in lipid profiles that either promote or inhibit viral replication, demonstrating the dual role of lipid metabolism in both facilitating viral entry and mounting an immune response. This complex relationship suggests that targeting lipid metabolism could serve as a therapeutic strategy against viral infections. For instance, the use of lipid inhibitors or dietary modifications, such as ketogenic diets, has been proposed as potential interventions to limit the severity of infections like COVID-19 (Ryu et al., 2020). Furthermore, the lipid composition of the viral envelope itself is crucial for the fusion process. Enveloped viruses, including human immunodeficiency virus (HIV) and influenza, rely on specific lipid interactions to mediate membrane fusion. For example, the exposure of phosphatidylserine (PtdSer) on the viral envelope and infected cells has been shown to facilitate viral entry by enhancing interactions with host cell receptors (Chua et al., 2019). This highlights the importance of lipid dynamics not only in the context of viral entry but also in the broader scope of viral pathogenesis and immune evasion.

Taken together, lipid-mediated membrane fusion is a fundamental mechanism of viral internalization that underscores the intricate relationship between viruses and host lipid metabolism. Viruses exploit host lipids at multiple stages of their life cycle, from entry to replication and assembly, while also manipulating lipid metabolic pathways to create a favorable environment for their propagation. Understanding these interactions opens new avenues for therapeutic interventions aimed at disrupting viral entry and replication by targeting lipid metabolism, potentially leading to novel antiviral strategies. As research continues to unveil the complexities of viral interactions with host lipids, it becomes increasingly clear that lipid metabolism plays a pivotal role in the pathogenesis of viral infections and the host’s immune response.

### 3.3 The relationship between lipid metabolism and viral assembly and release

The relationship between lipid metabolism and viral assembly is critical for the successful propagation of viruses. Enveloped viruses, in particular, rely on host lipids for the formation of their lipid bilayers during the budding process. This reliance on host lipids underscores the importance of lipid metabolism in the viral life cycle. For example, studies have shown that specific lipid species, such as sphingolipids and phospholipids, are enriched in viral particles, indicating their role in viral assembly (Shiota et al., 2023). In addition to the direct interaction between host lipids and viral assembly, the role of lipid droplets (LDs) in viral infections has garnered attention. LDs are cellular organelles that store neutral lipids and play a role in lipid metabolism and energy homeostasis. Recent studies indicate that LDs can be utilized by viruses to facilitate their replication and assembly, acting as reservoirs of lipids that are essential for viral life cycles (He et al., 2022). The accumulation of LDs during viral infections has been linked to altered immune responses, suggesting that these organelles may serve as both a resource for viral replication and a site for immune regulation (Soares et al., 2024). For instance, the dengue virus exploits LDs to enhance its replication and assembly, highlighting the critical role of lipid metabolism in the life cycle of flaviviruses (Zhang et al., 2017). Moreover, the release of viral particles from infected cells is also closely tied to lipid metabolism. The budding of enveloped viruses involves the incorporation of host lipids into the viral envelope, which is essential for the structural integrity and infectivity of the newly formed virions (Calistri et al., 2009). Dysregulation of lipid metabolism can lead to impaired viral release, thereby limiting the spread of the virus. Understanding the intricacies of how viruses hijack lipid metabolism for assembly and release can inform the development of antiviral therapies that target these processes, potentially reducing viral load and transmission.

### 3.4 Differential utilization of lipid metabolism by different viruses

**Figure 2:**
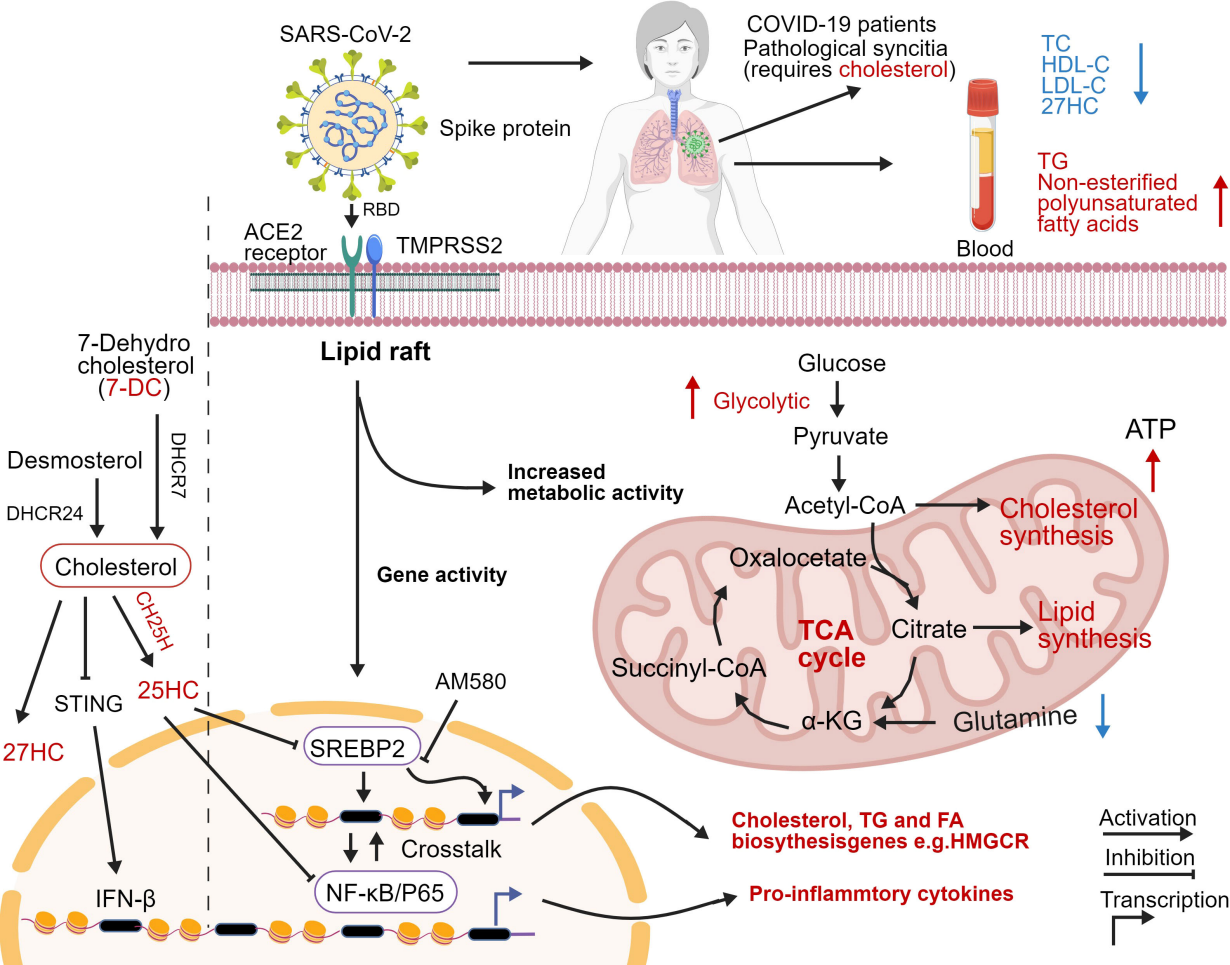
Overview of coronavirus infection. (Left of dotted line) Cholesterol metabolizing enzymes and metabolites act against viral infectivity. Red type represents cholesterol metabolizing enzymes or corresponding natural products which may be used as drug targets or directly to exert antiviral effects. (Right of dotted line) Cholesterol metabolism reprogramming and antiviral responses after viral infection. Cholesterol promotes pathological syncytial formation during SARS-COV-2 infection. Serum TC, TG, and non-esterified polyunsaturated fatty acid levels are altered in COVID-19 patients. SARS-CoV-2 infection increases glucose entry into the TCA cycle via increased pyruvate carboxylase expression and reduced oxidative glutamine metabolism, while maintaining reductive carboxylation. SREBP-dependent lipidomic reprogramming is a broad-spectrum antiviral target, AM580 strongly inhibits coronavirus replication by interacting with SREBP-2. COVID-19-activated SREBP-2 disturbs cholesterol biosynthesis, leading to a cytokine storm. Importantly, SREBP-2 activity is regulated by crosstalk between cholesterol consumption and NF-kB expression via several inflammatory response processes induced by SARS-CoV-2 infection. Red arrows: upregulation, blue arrows: downregulation. DHCR7, 7-dehydrocholesterol reductase; CH25H, cholesterol-25-hydroxylase; 25HC, 25-hydroxycholesterol; 27HC, 27-hydroxycholesterol; IFN-β, Interferon-β; TC, Total cholesterol; HDL-C, High-density lipoprotein cholesterol; LDL-C, low-density lipoprotein cholesterol; TG, Triglyceride; ATP, Adenosine triphosphate; SREBP-2, Sterol regulatory element-binding protein 2; AM580, a selective RARα agonist; HMGCR, 3-Hydroxy-3-Methylglutaryl Coenzyme A Reductase; NF-κB, Nuclear transcription factor-κB. Created with BioGDP.com.

Different viruses exhibit varying degrees of dependence on host lipid metabolism, reflecting their unique life cycles and pathogenic strategies. For instance, while enveloped viruses like SARS-CoV-2 and HCV extensively utilize host lipids for replication and assembly, other viruses may rely less on lipid metabolism or exploit it in different ways. The differences in lipid metabolism utilization can significantly impact the virulence and transmission dynamics of these viruses. For example, studies have shown that SARS-CoV-2 infection leads to significant alterations in the host lipid profile, with specific lipid species being upregulated or downregulated in response to the infection (Abdel-Megied et al., 2023). This manipulation of lipid metabolism not only supports viral replication but also modulates the host immune response, potentially contributing to the severity of COVID-19 (**Figure 2**). In contrast, viruses like the influenza virus may induce different metabolic changes, focusing on enhancing glycolysis and other metabolic pathways to support their replication (Keshavarz et al., 2020; Kleinehr et al., 2021). Additionally, the exploitation of lipid metabolism can vary based on the host’s metabolic state. For instance, in individuals with obesity, the altered lipid metabolism may facilitate increased viral replication and severity of infections, as seen in SARS-CoV-2 (Zhu et al., 2023). This highlights the importance of considering host factors when studying viral interactions with lipid metabolism.

A comparative examination of how diverse viruses manipulate host lipid metabolism reveals a fascinating interplay between conserved, shared mechanisms and distinct, virus-specific adaptations (**Table 1**). Enveloped viruses across different families converge on key host lipid pathways to fulfill fundamental stages of their life cycle. A prime shared strategy is the exploitation of cholesterol-rich lipid rafts for viral entry. Viruses as disparate as Influenza virus, SARS-CoV-2, and HIV utilize these membrane microdomains to concentrate receptors and co-facilitate membrane fusion, highlighting a common dependence on host membrane architecture for initial infection (Scheiffele et al., 1999; Chua et al., 2019; Dadhich and Kapoor, 2020). Furthermore, many viruses commonly hijack the SREBP pathway to fuel replication. Activation of SREBP-1, leading to upregulated fatty acid and cholesterol synthesis, is observed during infection by SARS-CoV-2, and HCV (Yu et al., 2012; Yuan et al., 2019; Ma et al., 2024). This shared tactic ensures a plentiful supply of lipid building blocks for nascent viral envelopes and replication compartments. Lastly, the co-option of Lipid Droplets (LDs) represents another widespread strategy. LDs serve as versatile platforms, providing lipids for membrane synthesis and sites for particle assembly. HCV, and SARS-CoV-2 induce and utilize LDs, with viral proteins like HCV NS5A, and SARS-CoV-2-associated proteins localizing to LD surfaces to facilitate virion formation (Filipe and McLauchlan, 2015; Zhang et al., 2017; Dias et al., 2020). Beyond these common themes, viruses exhibit specialized adaptations tailored to their unique replication niches and structures. HCV displays a particularly intricate relationship with LDs, where its non-structural protein NS5A not only localizes to LDs but also recruits viral replication complexes via interaction with host proteins like TIP47, effectively turning LDs into major hubs for both RNA replication and particle assembly—a level of integration less pronounced in other viruses (Vogt et al., 2013). In contrast, Influenza virus exhibits a distinct focus on modulating lipid metabolism to support its rapid replication cycle in the respiratory epithelium. It differentially activates mTORC1 and mTORC2 signaling to optimize cellular translation and metabolism for viral production, a nuanced regulation of the mTOR pathway not as prominently reported for all lipid-manipulating viruses (Kuss-Duerkop et al., 2017; Episcopio et al., 2019). Meanwhile, HIV-1 employs a unique strategy by leveraging its accessory protein Nef to perturb calcium homeostasis and lipid signaling, which not only promotes viral gene expression but also contributes to immune cell dysfunction and persistence—a strategy deeply tied to its pathogenesis in the immune system (Gokhale et al., 2021).

**Table 1:** Comparative overview of lipid metabolic strategies in selected viruses.

Virus	Shared strategies	Specific adaptations	References
SARS-CoV-2	Exploits lipid rafts for entry; Activates SREBP pathway; Induces Lipid Droplet formation.	Upregulates DGAT-1 and PPAR-γ for LD biogenesis; Alters phospholipid and sphingolipid profiles distinctly.	(Dadhich and Kapoor, 2020; Dias et al., 2020; Abdel-Megied et al., 2023)
Influenza virus	Utilizes lipid rafts for entry/budding; Modulates mTOR signaling.	Differential activation of mTORC1/mTORC2; PB1-F2 protein targets MAVS on mitochondria.	(Scheiffele et al., 1999; Episcopio et al., 2019; Kleinehr et al., 2021)
HCV	Activates SREBP & lipogenesis; Co-opts LDs for assembly.	NS5A protein intricately binds LDs via TIP47; Unique reliance on geranylgeranylation.	(Kapadia and Chisari, 2005; Vogt et al., 2013)
HIV-1	Uses lipid rafts for entry; Alters cholesterol metabolism.	Nef protein disrupts Ca^2+^ and lipid signaling; Promotes persistent infection in immune cells.	(Chua et al., 2019; Gokhale et al., 2021)

In conclusion, the differential utilization of lipid metabolism by various viruses underscores the complexity of virus-host interactions. By elucidating the specific mechanisms through which different viruses exploit lipid metabolism, researchers can identify potential therapeutic targets and develop strategies to mitigate viral infections effectively. Understanding these dynamics is crucial for advancing antiviral research and improving public health responses to viral outbreaks.

## 4 Lipid metabolism and host immune response interaction

### 4.1 The role of lipids in immune cell function

Lipid metabolism plays a crucial role in shaping immune cell function and responses. Immune cells, such as macrophages, T cells, and dendritic cells, rely on lipids not only as energy sources but also as signaling molecules that can modulate their activation, differentiation, and effector functions. Specifically, the balance between saturated and polyunsaturated fatty acids (PUFAs) influences macrophage polarization. For example, an increased ratio of omega-6 to omega-3 PUFAs can promote a pro-inflammatory M1 phenotype through enhanced prostaglandin E2 (PGE_2_) synthesis, while omega-3 PUFAs (e.g., DHA, EPA) favor an anti-inflammatory M2 phenotype via resolvin and protectin production, thereby affecting the overall immune response (Djuricic and Calder, 2021). In T cells, cholesterol-rich lipid rafts are essential for the assembly and signaling of the T cell receptor (TCR) complex. Modulating membrane cholesterol content, for instance via statins, can impair TCR clustering and downstream activation signals such as ZAP-70 phosphorylation, thereby affecting T cell proliferation and effector function (Zeyda and Stulnig, 2007). Furthermore, specific lipid species such as ceramide and sphingosine-1-phosphate (S1P) play opposing roles in immune cell survival. Ceramide promotes apoptosis in activated lymphocytes, whereas S1P supports cell survival and regulates lymphocyte egress from lymphoid organs, highlighting how sphingolipid metabolism finely tunes immune homeostasis (Thakkar et al., 2025). Dysregulation of lipid metabolism can lead to altered immune cell function, contributing to various diseases, including obesity (Ito, 2025), diabetes (Schoeler and Caesar, 2019), and autoimmune disorders (Han, 2025). Thus, understanding the intricate relationship between lipid metabolism and immune cell function is essential for developing therapeutic strategies targeting metabolic disorders and enhancing immune responses.

### 4.2 How lipid metabolites regulate antiviral immunity

Lipid metabolites have emerged as critical regulators of antiviral immunity, influencing both the innate and adaptive immune responses. For instance, certain lipid mediators, such as eicosanoids derived from arachidonic acid, are known to modulate the production of pro-inflammatory cytokines and chemokines in response to viral infections (Conti et al., 2020). Additionally, the accumulation of specific lipid species can enhance the antiviral state of cells. For example, 25-hydroxycholesterol (25-HC), a metabolite of cholesterol, has been shown to inhibit viral replication by modulating the expression of interferon-stimulated genes (ISGs) and enhancing the antiviral response (**Figure 2**). 25-HC exerts broad antiviral activity by inhibiting the membrane fusion of enveloped viruses (e.g., SARS-CoV-2, VSV) and by amplifying the expression of ISGs through modulating the activity of the cholesterol biosynthetic enzyme INSIG (Liu et al., 2013). Conversely, certain viruses can manipulate prostaglandin pathways to dampen immunity. For instance, SARS-CoV-2 viruses enhance the production of PGE_2_ via cyclooxygenase-2 (COX-2), which can suppress type I IFN production and cytotoxic T cell activity, facilitating viral immune evasion (Tian et al., 2021). Furthermore, the gut microbiota-derived metabolites, such as short-chain fatty acids, can influence the systemic immune response and enhance antiviral immunity by promoting the differentiation and function of T cells (Khawar et al., 2019). The interplay between lipid metabolism and immune signaling pathways is complex, as lipid metabolites can also serve as substrates for the synthesis of signaling molecules that activate immune responses. Understanding these regulatory mechanisms is crucial for developing novel antiviral therapies that harness the power of lipid metabolism to enhance immune responses against viral pathogens.

### 4.3 The impact of altered lipid metabolism on immunity in disease states

Alterations in lipid metabolism are increasingly recognized as significant contributors to immune dysfunction in various disease states. For instance, in obesity and metabolic syndrome, dysregulated lipid metabolism leads to an accumulation of pro-inflammatory lipids, which can activate immune cells and promote chronic inflammation (Hade et al., 2025). This chronic inflammatory state is associated with increased susceptibility to infections and impaired antiviral responses, as seen in patients with metabolic disorders. Similarly, in cancer, tumor cells often exhibit altered lipid metabolism, which not only supports their growth but also modulates the immune microenvironment, leading to immune evasion (Pascual and Benitah, 2024). The accumulation of specific lipid metabolites can suppress the activation and function of immune cells, further exacerbating the tumor’s ability to evade immune surveillance (Zhu et al). Conversely, targeting lipid metabolism to restore immune cell function has shown therapeutic promise. For instance, Yang et al. (2024) demonstrated that stilbene glycosides alleviate atherosclerosis partly by promoting lipophagy in dendritic cells, thereby modulating lipid accumulation and immune activity within the vascular wall. Moreover, In chronic viral infections like HIV, persistent antigen exposure coupled with dyslipidemia leads to T cell exhaustion. Exhausted T cells exhibit a distinct metabolic profile with impaired mitochondrial fatty acid β-oxidation and increased dependency on glycolysis, which is insufficient to support their long-term functionality. This metabolic impairment is reinforced by inhibitory receptor signaling (e.g., PD-1), creating a vicious cycle of metabolic and functional exhaustion (Kang and Tang, 2020). These examples demonstrate how disease-specific lipid metabolic shifts target precise immune mechanisms—from macrophage polarization and DC function to T cell metabolism and survival—culminating in compromised antiviral defense. These findings underscore the importance of lipid metabolism in shaping immune responses across various disease contexts, highlighting the potential for targeting lipid metabolic pathways as a therapeutic strategy to restore immune function and improve health outcomes.

## 5 Potential therapeutic strategies

### 5.1 Development of antiviral drugs targeting lipid metabolism

The interplay between viral infections and host lipid metabolism has garnered significant attention in recent years, particularly in the context of enveloped viruses such as SARS-CoV-2. Viruses exploit host lipids at various stages of their life cycle, including entry, replication, and egress. This dependency on host lipid metabolism presents a unique opportunity for the development of antiviral therapies that target lipid metabolic pathways. Research indicates that manipulating lipid metabolism can disrupt the viral lifecycle, potentially limiting viral replication and spread. For instance, preclinical studies have demonstrated that statins, which are primarily used to lower cholesterol levels, have been proposed as potential antiviral agents due to their ability to inhibit the synthesis of mevalonate, a precursor for cholesterol and other lipids essential for viral envelope formation (Dai et al., 2022). Additionally, studies have shown that specific lipid species, such as ceramides and sphingolipids, are crucial for viral entry and replication, suggesting that targeting these lipids could provide a therapeutic avenue (Beckmann and Becker, 2021; Dai et al., 2024). Furthermore, lipidomics approaches have identified distinct lipid profiles associated with viral infections, highlighting the potential for lipid-targeting drugs to serve as adjunctive therapies in the management of viral diseases (Yan et al., 2019). In the clinical context, observational studies and retrospective analyses have explored the repurposing of existing lipid-modulating drugs. For example, some clinical studies during the COVID-19 pandemic investigated the association between statin use and disease outcomes, though results have been mixed and causal relationships are not firmly established (Kow and Hasan, 2020; Zhang et al., 2020). These observations highlight the need for well-designed prospective clinical trials. More speculative, hypothesis-driven strategies include the design of novel small molecules that specifically inhibit viral exploitation of host lipid synthesis pathways (e.g., targeting SREBP activation) or the development of lipid nanoparticle-based delivery systems to selectively interfere with viral assembly. The development of such novel antiviral drugs that target lipid metabolism holds promise for enhancing the efficacy of existing therapies and providing new treatment options, but their transition to clinical application requires rigorous validation. Overall, the development of antiviral drugs that target lipid metabolism holds promise for enhancing the efficacy of existing antiviral therapies and providing novel treatment options for viral infections (**Table 2**).

**Table 2:** Drugs targeting lipid pathways currently in clinical trials for COVID-19.

Drug(s)	Target	Rationale for use in COVID-19	ClinicalTrials.gov Identifier/Reference
Ibuprofen	COX-1 and COX-2	COX-2 inhibition is anti-inflammatory and improves survival in preclinical models of viral infection. Inhibition of PGE2 synthesis enhances the type I interferon response to viral infection.	NCT04334629, NCT04382768
Celecoxib	COX-2	COX-2 inhibition is anti-inflammatory and improves survival in preclinical models of viral infection. Inhibition of PGE2 synthesis enhances the type I interferon response to viral infection.	NCT04488081
Low-dose aspirin	Platelet COX-1	Inhibition of TxA2 synthesis decreases platelet aggregation, which may prevent thrombotic complications of COVID-19.	NCT04365309, NCT02735707, NCT04703608, NCT04483960, NCT04333407, NCT04324463, NCT04381936, NCT04498273, NCT04368377, NCT04363840, NCT04466670, NCT04808895, NCT04937088, NCT04768179, NCT04410328
Epoprostenol and iloprost	IPr	Prostacyclin analogs promote vasodilation in the pulmonary vasculature, which improve inflammation and oxygenation in COVID-19 patients with ARDS.	NCT04705597, NCT04420741, NCT04445246
BGE-175	DPr1	Inhibition of DPr1 signaling enhances the adaptive immune response to viral infection in preclinical models	NCT04705597
Montelukast and Zafirlukast	CysLT1R	CysLT1R inhibition is anti-inflammatory and decreases airway hyper-responsiveness after pulmonary viral infection.	NCT04871828, NCT04718285, NCT04695704, NCT04389411, NCT04714515
EPA, DHA, and icosapent ethyl	N/A	Omega-3 fatty acids have anti-inflammatory effects.	NCT04505098, NCT04412018, NCT04460651, NCT04957940, NCT04658433, NCT04495816, NCT04647604, NCT04483271;
Statins	HMG-CoA Reductase	Inhibition of cholesterol synthesis may deplete cholesterol in lipid rafts. Statins have pleiotropic anti-inflammatory effects and have been associated with improved outcomes in patients with viral pneumonia.	NCT04472611, NCT04904536, NCT04359095, NCT04801940, NCT04380402, NCT04466241, NCT04952350, NCT04900155, NCT02735707, NCT04333407
Fenofibrate	PPAR-α	PPAR-α agonism may reverse alterations in lipid metabolism induced by SARS-CoV-2 and block viral replication.	NCT04517396, NCT04661930
Evolocumab	PCSK9	PCSK9 loss-of-function genetic variants have been associated with a decrease in inflammatory cytokine response and improved survival in septic shock patients.	NCT04941105
Lerodalcibep	PCSK9	Block PCSK9-mediated LDL receptor degradation, indirectly reducing viral attachment to host cells and dampening cytokine storms.	NCT04797104
Opaganib	SK2	SK inhibition suppresses viral replication and inhibits the hyperinflammatory response to viral infection.	NCT04467840, NCT04414618
Ozanimod	S1P1 and S1P5	Activation of S1P signaling restrained cytokine storm, reduced lung pathology, and improved survival in preclinical models of viral infection.	NCT04405102
Icosapent Ethyl	Omega-3 Polyunsaturated Fatty Acids (PUFAs)	Reduces pro-inflammatory cytokines (e.g., TNF-α) and platelet aggregation .	NCT04333899
Niacin(Vitamin B3)	N/A	Modulates HDL composition, potentially blocking viral attachment to ACE2 receptors .	NCT04435233

### 5.2 Modulating lipid metabolism to enhance immune response

The modulation of lipid metabolism has emerged as a critical strategy for enhancing the immune response against viral infections. Lipids play multifaceted roles in immune cell function, influencing processes such as cell signaling, membrane integrity, and energy metabolism. For instance, preclinical evidence indicates that alterations in lipid profiles can affect the activation and differentiation of immune cells, including T cells and macrophages, which are pivotal in the antiviral immune response (Hubler and Kennedy, 2016). Research has shown that specific lipid mediators, such as eicosanoids and sphingolipids, can modulate inflammation and immune cell activity, suggesting that lipid metabolism is intricately linked to the regulation of immune responses (Garcia et al., 2023). Moreover, clinical observations and emerging intervention studies suggest potential avenues. For example, interventions aimed at enhancing lipid metabolism, such as dietary modifications or pharmacological agents (e.g., AMPK activators) have been explored for their immunomodulatory effects in metabolic and inflammatory conditions, with implications for viral infections (O’Neill et al., 2016; Molendijk et al., 2019). Small-scale human studies have proposed that ketogenic diets, which promote the utilization of fatty acids for energy, have been proposed as a means to enhance the immune response by shifting the metabolic profile of immune cells (Patikorn et al., 2023). Future, more speculative strategies involve precisely reprogramming the lipid metabolism of specific immune cell subsets (e.g., via adoptive cell therapy or targeted metabolic modulators) to enhance their antiviral capacity. Another hypothesis-driven approach is the use of specialized pro-resolving lipid mediators to actively resolve virus-induced inflammation without compromising viral clearance. These strategies are largely conceptual and require extensive preclinical investigation. Thus, strategically modulating lipid metabolism could serve as a powerful tool for boosting the immune response and improving outcomes in viral infections.

### 5.3 Combination therapy: lipid metabolism modulation with conventional antiviral treatment

The integration of lipid metabolism modulation with conventional antiviral therapies represents a promising approach to enhancing treatment efficacy against viral infections. Combination therapy has the potential to address the multifaceted nature of viral pathogenesis, where both viral replication and host immune responses are targeted simultaneously. Preclinical research provides a strong rationale for this approach. Studies in cell culture and animal models have demonstrated that the co-administration of lipid-modulating agents (e.g., statins, fatty acid synthase inhibitors) with established antiviral drugs can lead to synergistic effects, improving viral clearance and reducing disease severity compared to monotherapy (Gong et al., 2021; Zapatero-Belinchón et al., 2021). Clinical exploration of such combinations is in its early stages. Some clinical trials have begun to evaluate the safety and efficacy of adding statins or other metabolic modulators to standard antiviral regimens for infections like COVID-19 (Cariou and Goronflot, 2021). Preliminary results are promising but not yet conclusive, underscoring the necessity for larger, phase III trials to determine clinical benefit and optimal combinations. A more forward-looking, hypothesis-driven framework involves designing personalized combination therapies based on the patient’s metabolic profile (e.g., obesity, dyslipidemia) and the specific lipid dependencies of the infecting virus. This could involve integrating metabolomic profiling into clinical decision-making to guide adjunctive lipid-targeting therapy. This approach remains speculative and hinges on advances in point-of-care diagnostics and a deeper understanding of individual metabolic-viral interactions. This combined approach not only targets the virus directly but also optimizes the host’s immune response, thereby addressing the challenges posed by viral infections more comprehensively. As research continues to elucidate the intricate relationship between lipid metabolism and viral pathogenesis, the development of combination therapies that leverage this knowledge will be crucial for advancing antiviral treatment strategies.

## 6 Future research directions

### 6.1 Interaction studies between lipid metabolism and novel viral strains

The interplay between lipid metabolism and viral infections has garnered significant attention, particularly in the context of novel viral strains such as SARS-CoV-2. Viruses have evolved mechanisms to exploit host lipid metabolism for their replication and propagation. For instance, enveloped viruses like coronaviruses utilize host lipids during various stages of their lifecycle, including viral entry and replication. Recent studies indicate that dyslipidemia may exacerbate the severity of COVID-19, suggesting that targeting lipid metabolism could offer therapeutic benefits against such viral infections (Fabre et al., 2022). Furthermore, alterations in lipid profiles during viral infections can significantly affect membrane properties, which are crucial for viral entry and replication (Havranek et al., 2021). Future research should focus on elucidating the specific lipid species that are manipulated by viruses and the pathways through which these interactions occur. For example, the role of sphingolipids and glycerophospholipids in the viral lifecycle needs to be further explored, as these lipids have been shown to be significantly altered during viral infections (Martín-Acebes et al., 2014; Strating and van Kuppeveld, 2017). Additionally, understanding the mechanisms by which viruses hijack lipid metabolism could lead to the identification of novel therapeutic targets. For instance, the use of lipid inhibitors or supplementation strategies could potentially limit viral replication by restricting the availability of essential lipids to the virus (Pérez and Carrasco, 1991; Alketbi et al., 2021). Moreover, the relationship between obesity, lipid metabolism, and viral infections presents a compelling area for investigation. Adipocytes may serve as reservoirs for viral replication, particularly in individuals with obesity, where altered lipid metabolism can facilitate viral entry and replication (Al-Benna). This highlights the need for comprehensive studies that examine how metabolic disorders influence viral pathogenesis and the potential for therapeutic interventions that target lipid metabolism in the context of viral infections.

### 6.2 Applications of metabolomics in viral infection research

Metabolomics, the comprehensive study of metabolites within biological systems, has emerged as a powerful tool in understanding the metabolic changes that occur during viral infections. By profiling metabolites, researchers can gain insights into the host’s metabolic response to viral pathogens and the alterations that facilitate viral replication. For instance, studies have shown that influenza virus infection leads to significant changes in cellular metabolic pathways, including increased glycolysis and altered lipid metabolism (Chen et al., 2023). These metabolic alterations are not merely byproducts of infection but are actively manipulated by viruses to create an environment conducive to their replication. The application of metabolomics in viral research can provide a detailed understanding of the metabolic signatures associated with different viral infections. For example, distinct metabolic profiles have been identified in patients with COVID-19, which correlate with disease severity and can serve as potential biomarkers for diagnosis and treatment (Bennet et al., 2022). Furthermore, the integration of metabolomics with other omics technologies, such as proteomics and genomics, can enhance our understanding of the complex interactions between viruses and host metabolism (Gameli et al., 2023). Future studies should focus on utilizing advanced metabolomic techniques to identify specific metabolites that are indicative of viral infections and their severity. This could lead to the development of targeted therapeutic strategies that modulate metabolic pathways to enhance the host’s antiviral response. Additionally, understanding the role of metabolites in immune modulation during viral infections could open new avenues for therapeutic interventions aimed at boosting the immune response against viral pathogens (Xu et al).

### 6.3 Translational medicine research: lipid metabolism and pandemic control

The intersection of lipid metabolism and viral infections has significant implications for translational medicine, particularly in the context of pandemic control. As evidenced by the COVID-19 pandemic, understanding the metabolic pathways involved in viral pathogenesis is crucial for developing effective treatment strategies. Lipid metabolism plays a pivotal role in the immune response to viral infections, and alterations in lipid profiles can influence disease outcomes (Albert et al., 2022). Research in translational medicine should focus on identifying lipid-based therapeutic targets that can be utilized to mitigate the effects of viral infections. For instance, the use of statins, which are known to modulate lipid metabolism, has been proposed as a potential therapeutic strategy against SARS-CoV-2 (Abu-Farha et al., 2020). Additionally, dietary interventions that alter lipid metabolism, such as ketogenic diets, may offer novel approaches to enhance the host’s resilience against viral infections (Alketbi et al., 2021). Furthermore, the role of lipid mediators in regulating the immune response during viral infections presents an opportunity for therapeutic development. Understanding how specific lipids influence inflammation and immune cell function could lead to the identification of new anti-inflammatory agents that can be used in conjunction with antiviral therapies.

Collectively, future research directions should prioritize the exploration of lipid metabolism as a therapeutic target in viral infections, with an emphasis on translational approaches that can be rapidly implemented during pandemics. This includes investigating the potential of lipid-based therapies, dietary modifications, and the modulation of lipid mediators to enhance the host’s immune response and improve clinical outcomes in viral infections.

## 7 Discussion

The intricate relationship between lipid metabolism, viral infections, and antiviral immunity is an area of growing interest and significance in the field of medical research. As we have discussed throughout this review, the interplay between these biological systems is not only fundamental to our understanding of viral pathogenesis but also opens up new avenues for therapeutic interventions. Lipid metabolism plays a crucial role in cellular function, and its dysregulation can lead to altered immune responses and increased susceptibility to viral infections. The association between lipid profiles and viral pathogenesis highlights the need for a multidisciplinary approach that integrates insights from virology, immunology, and metabolic research. By examining how different lipids influence viral entry, replication, and the host’s immune response, researchers can identify potential therapeutic targets that could enhance antiviral strategies. Moreover, the immune system’s response to viral infections is significantly influenced by lipid metabolism. For instance, certain lipids serve as signaling molecules that can modulate immune cell function and inflammation (Varga and Nagy, 2008; Cooper et al., 2024). Understanding how viruses exploit host lipid pathways to evade immune detection and response is critical for developing effective vaccines and antiviral therapies. As such, future studies should focus on elucidating these complex interactions, providing a more nuanced view of how lipid metabolism can be manipulated to bolster antiviral immunity.

The impact of these findings extends beyond basic research; they have practical implications for clinical applications. The potential for lipid-based therapies to enhance vaccine efficacy or to serve as adjuncts to antiviral drugs is an exciting prospect. By leveraging our understanding of lipid metabolism, we may be able to devise strategies that not only prevent viral infections but also improve recovery outcomes in infected patients. This could be particularly pertinent in the context of emerging viral threats, where rapid and effective responses are paramount. However, balancing the diverse perspectives in this research field poses challenges. Different studies may yield varying results based on methodologies, specific viral strains examined, and host models utilized. Therefore, it is crucial for researchers to adopt a collaborative approach, integrating findings from different studies to form a more cohesive understanding of the lipid-viral interaction. Establishing standardized protocols and utilizing advanced technologies, such as lipidomics and systems biology, can help reconcile discrepancies and foster a more unified approach to exploring these complex relationships. While recent reviews have highlighted the role of lipid metabolism in different virus infection (Girdhar et al., 2021; Dai et al., 2022), our manuscript offers a broader and more integrative perspective. Specifically, this review provides a comprehensive analysis of lipid metabolism beyond cholesterol, encompassing fatty acid synthesis, degradation, and signaling, and details their collective manipulation by a diverse range of viruses (including but not limited to coronaviruses, influenza, HCV, and HIV) across all stages of the viral life cycle. Furthermore, we place greater emphasis on the bidirectional interaction between viral-induced lipid metabolic reprogramming and the host’s antiviral immune response, systematically exploring how dysregulated lipid metabolism contributes to immune dysfunction in disease states. Finally, we synthesize potential therapeutic strategies targeting lipid pathways and propose future research directions focused on emerging viral strains, metabolomics applications, and pandemic control. Therefore, this review uniquely positions lipid metabolism as a central, dynamic host-pathogen interface, offering a holistic and mechanistic framework for developing broad-spectrum antiviral interventions.

## 8 Conclusion

The exploration of lipid metabolism’s role in viral infections and antiviral immunity is a promising frontier in medical research. This review underscores the importance of continued investigation into the mechanisms underlying these interactions, as they hold the potential to inform novel therapeutic strategies. Future research should aim to harmonize the various findings within the field, fostering collaboration among scientists from diverse disciplines. Therefore, we can enhance our understanding of viral pathogenesis and immunity, ultimately leading to the development of innovative approaches to prevent and treat viral infections. As we advance our knowledge in this area, the ultimate goal remains clear: to leverage the insights gained from lipid metabolism to improve public health outcomes and combat the ever-evolving challenges posed by viral pathogens.
